# Biventricular repair in low-weight patient with interrupted aortic arch and aortic atresia

**DOI:** 10.1186/s43044-024-00516-z

**Published:** 2024-07-04

**Authors:** Ilya A. Soynov, Alexey N. Arkhipov, Serezha N. Manukian, Yuriy Y. Kulyabin, Evgeniy Kobelev, Oksana Y. Malakhova, Alexey V. Voitov, Olga A. Suzdalova

**Affiliations:** Department of Pediatric Cardiac Surgery, E. Meshalkin National Medical Research Center, Ministry of Health of Russian Federation, Rechkunovskaya Str. 15, Novosibirsk, Russia 630055

**Keywords:** Congenital heart disease, Aortic atresia, Bilateral patent ductus arteriosus

## Abstract

**Background:**

Aortic atresia with ventricular septal defect is a very rare congenital cardiac anomaly, especially in combination with aortic arch interruption. It is always challenging to choose the optimal treatment tactics for such patients. One of the possible types of intervention is the Yasui procedure. There are only 19 reported cases in the literature of aortic atresia with interruption of the aortic arch type B or C, and not a single clinical case of type A.

**Case presentation:**

The proband was a 2-day-old boy with diagnosis: aortic atresia with a ventricular septal defect and interruption of the aortic arch type B. The child underwent a Yasui procedure without serious postoperative complications and with good long-term result.

**Conclusions:**

The Yasui procedure in patients with aortic atresia and interrupted aortic arch can be performed with minimal complications, even in low-weight patients.

## Background

Aortic atresia with ventricular septal defect is an exceptionally rare congenital heart defect, occurring in just 2 out of every 100,000 live births [1]. The morphological basis of aortic atresia with ventricular septal defect is a result of severe malalignment of the conal septum, leading to aortic valve atresia and a large ventricular septal defect, allowing for the normal development of the left ventricle and the mitral valve. A highly uncommon morphological form of aortic valve atresia with ventricular septal defect is associated with interruption of the aortic arch [2]. In this type of heart defect, the sole source of blood supply to the brain and heart is the aortopulmonary window. Additional blood supply can occur through collateral vessels from the second aortic arch, an aberrant subclavian artery, bilateral arterial duct, or the descending aorta [2–4].

We present a rare clinical case of biventricular treatment in a low-weight patient with aortic atresia combined with interruption of the aortic arch type B and bilateral patent ductus arteriosus.

### Case presentation

**Fig. 1:**
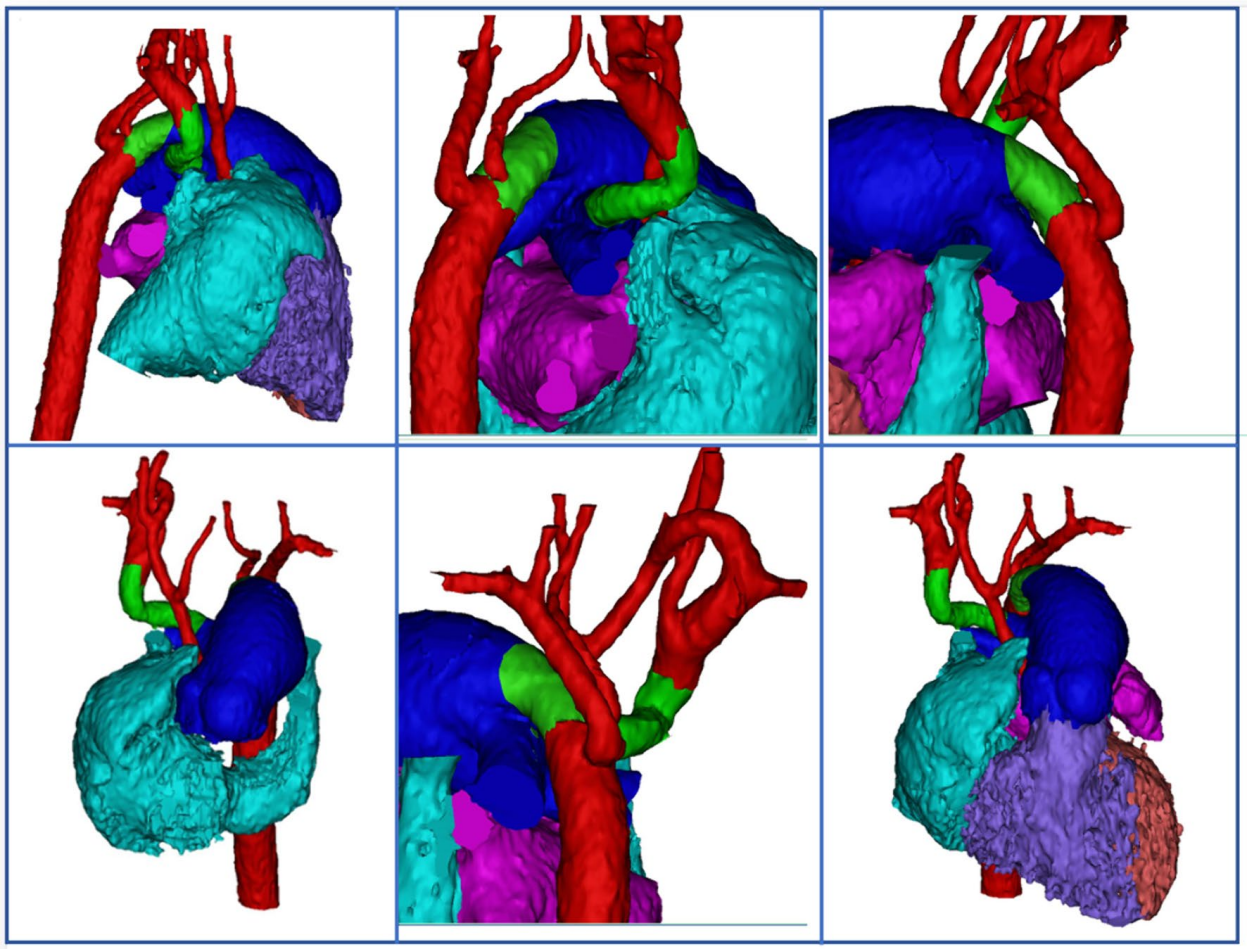
CT scan before the surgery: aortic atresia with a ventricular septal defect and interruption of the aortic arch type B

A 2-day-old male infant weighing 2.1 kg was admitted to our clinic with a preliminary diagnosis of hypoplastic left heart syndrome. The child received a PGE1 infusion at a dosage of 5 ng/kg/min. According to the echocardiogram, both ventricles were formed up to the apex of the heart, the contractile ability of both ventricles was normal (left ventricular ejection fraction 76%, right ventricular ejection fraction 42%), the mitral valve measures 10 mm (Z score − 0.54), the ascending aorta measures 2 mm (Z score − 9.3), the interventricular septal defect measures 10 mm, and the pulmonary artery trunk measures 10 mm. There was aortic valve atresia and an interruption of the aortic arch type B. The descending aorta was supplied by a 5-mm left arterial duct, and the ascending aorta by a 4 mm right arterial duct. A computed tomography (CT) scan was performed, and the diagnosis was confirmed: aortic atresia with a ventricular septal defect and interruption of the aortic arch type B (Fig. 1).

The special feature of the blood supply was bilateral arterial ducts, separately supplying the upper and lower halves of the body. On the 5th day, the child underwent a Yasui procedure. Monitoring of arterial pressure was carried out in the right radial and femoral arteries. Cerebral oximetry was performed using the INVOS 5100 device (Somanetics, USA) throughout the procedure. Extracorporeal circulation was conducted using Dideco Lilliput I systems (Sorin, Italy). The primary filling volume of the extracorporeal circuit was 200–250 ml and included donor erythrocyte mass (to maintain a hematocrit of at least 30%), 20% albumin (5 ml/kg), sodium bicarbonate 4%, mannitol, aminocaproic acid, and heparin. Access to the heart and main vessels was achieved through midsternotomy. Arterial cannulation was performed through the right arterial duct (used as a shunt for cerebral perfusion), while the second cannula was placed through the posterior leaflet of the pericardium into the descending aorta. Cannulas of equal diameter 8 Fr were used for symmetrical perfusion. A venous cannula was placed in the right atrium. We maintained a "warm" temperature regime at 32 °C with a full-flow rate of 150 ml/kg/min, which was evenly distributed between the two arterial lines. The gas composition of blood was maintained in PH–stat mode and monitored every 30 min. Cardioplegia was delivered into the aortic root through a DeBakey needle using Bretschneider solution (Custodiol Dr. Franz Kohler Chemie, Alsbach-Hahnlein, Germany) at a dosage of 50 ml/kg. Perfusion of the brain and internal organs was achieved through double cannulation of the aorta. Initially, the left arterial duct was ligated at the aortic and pulmonary ends, after which the duct was transected to mobilize the descending aorta. The right arterial duct was also ligated at the pulmonary end before arterial cannulation, and then, the duct was transected.

**Fig. 2:**
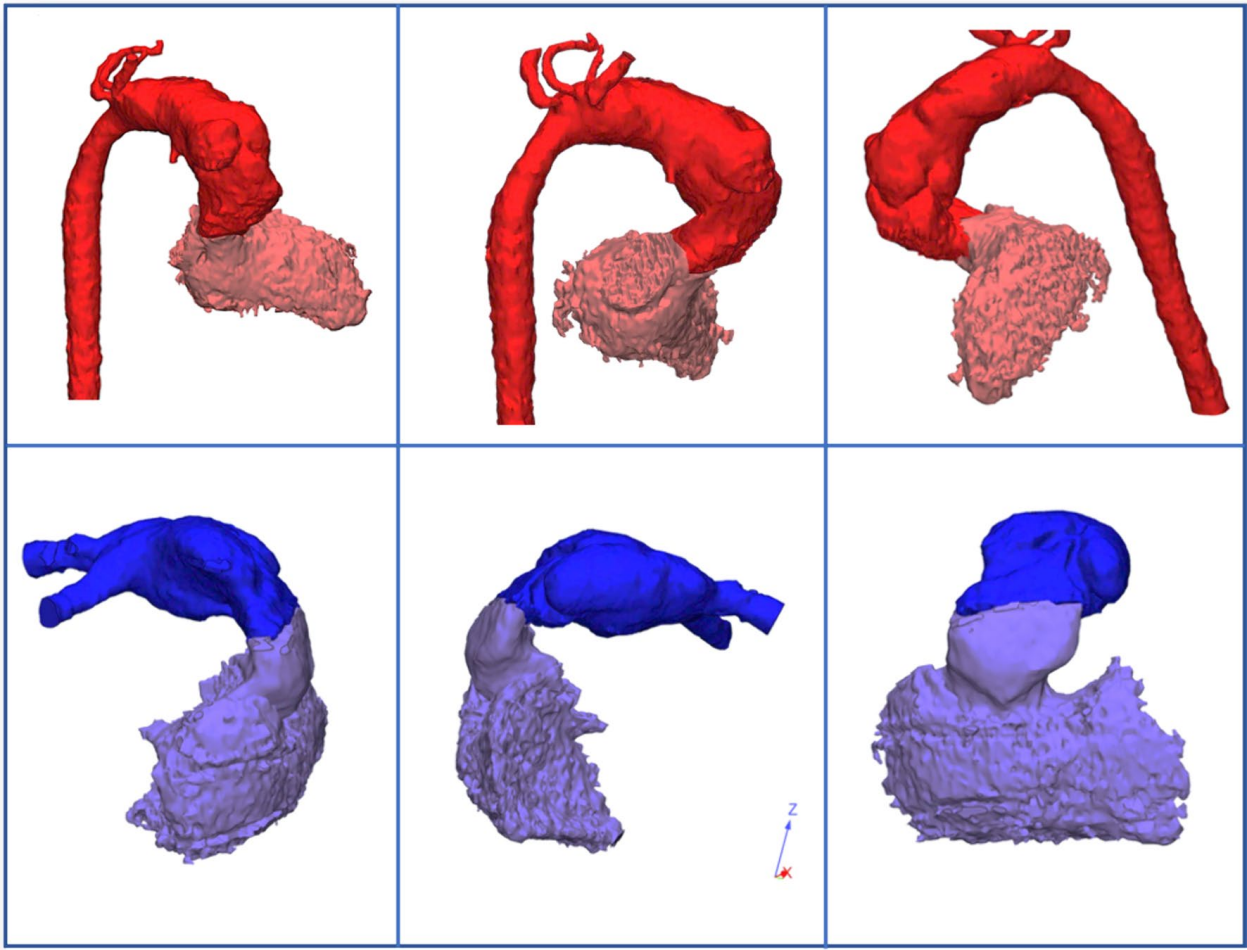
CT scan after 3 months: no deformities in the aorta and pulmonary arteries

The surgical details involved creating a xenopericardial tunnel from the left ventricle through the ventricular septal defect into the pulmonary artery. The creation of a Damus–Kaye–Stansel anastomosis was performed. The aortic arch was formed by anastomosing the descending aorta to the ascending aorta, simultaneously reconstructing the aortic arch using a patch from a pulmonary homograft. The final stage involved establishing the outflow tract from the right ventricle to the pulmonary artery using a 10-mm venous homograft. Cerebral and visceral perfusion was achieved through double cannulation of the aorta. The aortic occlusion time was 90 min. The duration of cardiopulmonary bypass was 145 min. After weaning from cardiopulmonary bypass, inotropic support included 0.03 mcg/kg/min of epinephrine and 0.025 mcg/kg/min of norepinephrine. The surgical chest diastasis lasted 2 days. Cardiac inotropic support was required for 4 days. Mechanical ventilation was needed for 6 days. On the 7th day, the child was transferred from the intensive care unit (ICU). The duration of hospitalization was 25 days. The child was discharged with good contractile function of both ventricles and no gradients at the LV outflow tract and aorta. A follow-up CT scan after 3 months showed no deformities in the aorta and pulmonary arteries (Fig. 2).

## Discussion

Aortic atresia with ventricular septal defect is a very rare congenital cardiac anomaly, especially in combination with aortic arch interruption due to possible impairment of coronary perfusion [3]. Currently, there are 19 reported cases in the literature of aortic atresia with interruption of the aortic arch type B or C, and not a single clinical case of type A, possibly due to the peculiarities of coronary and cerebral blood flow [2–8]. The sources of coronary and cerebral blood flow in cases of interrupted aortic arch are most commonly aortopulmonary windows, second aortic arches, aberrant subclavian arteries with retrograde blood flow through the circle of Willis, and rarely bilateral arterial ducts and collateral blood supply [2–4]. Therefore, type A and type B cannot survive without an association with aberrant subclavian, aortopulmonary window, or bilateral arterial ducts [3].

Embryologically, this defect develops between the fifth and seventh week of pregnancy, where severe malalignment of the conal septum in utero results in the absence of antegrade blood flow through the ascending aorta, leading to aortic valve atresia and hypoplasia of the ascending aorta. Deviation of the conal septum to the left and back leads to the appearance of a large interventricular defect in the conoventricular or perimembranous area, allowing both ventricles of the heart to develop [3, 9].

A distinguishing feature of our patient was the low patient weight. In previously described studies, authors preferred to perform palliative interventions [2, 10]. In 1987, Hasato Yasui first developed and performed biventricular correction in a patient with severe subaortic obstruction and a ventricular septal defect beneath the aortic valve [11]. The operation involves connecting the roots of the aorta and pulmonary artery to create a single outflow opening with aortic arch plastic surgery, closure of the ventricular septal defect, and the formation of a conduit between the right ventricle and the pulmonary artery (the Norwood-Rastelli procedure) [1].

In our case, we performed the Yasui procedure. We did not observe specific complications in the early postoperative period, except for respiratory insufficiency, which required prolonged mechanical ventilation for 6 days. In cases of aortic valve atresia with double outlet right ventricle (DORV), the surgical solution is usually straightforward as the left ventricular size and mitral valve are typically normal, with the procedure of choice being the Yasui operation, as was the case in our situation [12]. However, in cases of left ventricular outflow tract (LVOT) stenosis with DORV, the decision between traditional repair and the Yasui procedure may be challenging as there are no universally accepted criteria[1, 12]. Currently, there are several useful rules that can help determine the most appropriate corrective approach: Kanter and colleagues [13] suggest that the LVOT should be < 4 mm in newborn patients, while Nakano and colleagues [14] propose that the LVOT should be less than the body weight in kg + 1 mm. Z score values can also be useful in surgical decision-making. Abarbanell and colleagues found that a z score < − 2.5 indicates a risk of recurrent LVOT narrowing with standard repair and increases the risk of reoperation relative to the Yasui procedure by 2.7 times [15]. When performing the Yasui procedure, it is always necessary to take into account the size and localization of the DORV, the adequacy of LV inflow, the degree of LVOT hypoplasia, and the suitability of the pulmonary valve [12]. These markers help minimize early mortality and complications. The outcomes of the Yasui operation are quite well studied, with current early mortality rates not exceeding 15% [1]. Mortality often depends on the surgical treatment strategy. Carrillo and colleagues have shown that primary biventricular correction is preferable to staged treatment, as it reduces early postoperative mortality [16]. Genetic diseases, often associated with aortic arch interruption, can influence postoperative outcomes [17]. One specific complication is complete heart block, occurring in 5–10% of cases and potentially complicating the early postoperative course [16, 18]. In our case, we did not observe specific complications in the early postoperative period, except for respiratory insufficiency, which required prolonged mechanical ventilation for 6 days. In the long term, there are two complications that impact the frequency of reoperations: stenosis of the conduit between the pulmonary artery/left atrium and stenosis of the left ventricle/aorta tunnel [19]. The frequency of LVOT stenosis may depend on the size of the DORV relative to the pulmonary valve during the primary operation. The frequency of reoperations may increase up to 20% if the DORV was smaller than the pulmonary valve [13, 16, 20]. Some authors suggest that routine enlargement of the DORV will reduce the risk of subsequent LVOT stenosis. However, most studies have shown that there is no need to enlarge the DORV when it is already large, as this does not reduce the frequency of LVOT stenosis but increases the incidence of complete heart block [20]. The choice of material for tunnel formation did not show statistical differences in many studies, so in our case, we used xenopericardium [13, 19–21]. The replacement of the pulmonary artery/left atrium conduit is the most common reason for reoperations in the Yasui procedure [18]. According to Greene, half of all patients require conduit replacement within 5 years [19]. The choice of conduit significantly affects the frequency of reoperations, with pulmonary homografts being preferred and considered the gold standard among conduits [22]. In our case, we implanted a 10-mm venous homograft in a small child. This allowed us to avoid a wide right ventriculotomy and implant a conduit with a valve to reduce the risk of tricuspid insufficiency.

An alternative to the Yasui procedure may be the Ross-Konno or Norwood procedure. If a child is following a univentricular hemodynamics pathway, where the initial operation is the Norwood procedure, by the age of 5, the child will have undergone at least 3 surgeries, and the long-term survival rate is around 76–80% [23, 24]. If a newborn patient undergoes the Ross-Konno procedure, early mortality rates can reach 33% compared to the Yasui procedure, which appears to be more preferable [1, 18, 19]. Therefore, we chose the Yasui procedure for a safer biventricular correction strategy.

## Conclusions

The Yasui procedure in patients with aortic atresia and interrupted aortic arch can be performed with minimal complications, even in low-weight patients.

## Data Availability

Not applicable.

## Abbreviations

CT: Computed tomography
ICU: Intensive care unit
